# COVID-19-induced vascular angiopathy: CTPA signs in critically ill patients other than acute pulmonary embolism and high-lung opacity scores

**DOI:** 10.1186/s43055-021-00491-4

**Published:** 2021-04-19

**Authors:** Mohamed Hossameldin khalifa, Ahmed Samir, Ayman Ibrahim Baess, Sara Samy Hendawi

**Affiliations:** 1 Department of Radio-diagnosis and Intervention, Faculty of Medicine, University of Alexandria, Alexandria, Egypt; 2 Department of Chest Diseases, Faculty of Medicine, University of Alexandria, Alexandria, Egypt

**Keywords:** COVID-19, Vascular angiopathy, Artificial Intelligence

## Abstract

**Background:**

Vascular angiopathy is suggested to be the major cause of silent hypoxia among COVID-19 patients without severe parenchymal involvement. However, pulmonologists and clinicians in intensive care units become confused when they encounter acute respiratory deterioration with neither severe parenchymal lung involvement nor acute pulmonary embolism. Other radiological vascular signs might solve this confusion. This study investigated other *indirect vascular angiopathy signs* on CT pulmonary angiography (CTPA) and involved a novel statistical analysis that was performed to determine the significance of associations between these signs and the CT opacity score of the pathological lung volume, which is calculated by an artificial intelligence system.

**Results:**

The study was conducted retrospectively, during September and October 2020, on *73 patients with critical COVID-19* who were admitted to the ICU with progressive dyspnea and low O_2_ saturation on room air (PaO_2_ < 93%). They included 53 males and 20 females (73%:27%), and their age ranged from 18 to 88 years (mean ± SD=53.3 ± 13.5). *CT-pulmonary angiography* was performed for all patients, and an *artificial intelligence system* was utilized to quantitatively assess the diseased lung volume. The radiological data were analyzed by three expert consultant radiologists to reach consensus. A low CT opacity score (≤10) was found in 18 patients (24.7%), while a high CT opacity score (>10) was found in 55 patients (75.3%). Pulmonary embolism was found in 24 patients (32.9%); three of them had low CT opacity scores. Four other indirect vasculopathy CTPA signs were identified: (1) pulmonary vascular enlargement (57 patients—78.1%), (2) pulmonary hypertension (14 patients—19.2%), (3) vascular tree-in-bud pattern (10 patients—13.7%), and (4) pulmonary infarction (three patients—4.1%). There were no significant associations between these signs and the CT opacity score (0.3205–0.7551, all >0.05). Furthermore, both pulmonary vascular enlargement and the vascular tree-in-bud sign were found in patients without pulmonary embolism and low CT-severity scores (13/15–86.7% and 2/15–13.3%, respectively).

**Conclusion:**

Pulmonary vascular enlargement or, less commonly, vascular tree-in-bud pattern are both indirect vascular angiopathy signs on CTPA that can explain the respiratory deterioration which complicates COVID-19 in the absence of severe parenchymal involvement or acute pulmonary embolism.

## Background

By March 2020, COVID-19 was declared a pandemic by the WHO. Since then, there has been increased concern about the appropriate diagnostic methods, including PCR and CT [1, 2]. The recent guidelines from the “American Society of Radiology and Centers for disease control” advise that imaging modalities should be reserved for areas in which RT-PCR is not readily available or for moderate/severe cases to detect complications [3, 4].

The most commonly reported CT imaging findings in patients with COVID-19 were parenchymal lung changes, including ground-glass opacities, consolidations, the crazy paving pattern, and fibrotic changes [5]. However, these morphological changes could not explain the sudden deterioration in patients’ respiratory condition later in the disease with or without acute respiratory distress syndrome (ARDS). Recent research has attributed this to the associated pulmonary vasculopathy, endothelial damage, and altered vascular response to hypoxia with or without hypercoagulability and pulmonary embolism (PE) [6].

The European Society of Radiology and the European Society of Thoracic imaging recommended the use of CT pulmonary angiography (CTPA) when non-enhanced CT cannot explain the severity of the respiratory failure [7].

The pulmonologists and clinicians in intensive care units have been confused by the development of acute respiratory deterioration in COVID-19 patients with neither severe parenchymal lung involvement nor acute pulmonary embolism. Other radiological signs may be able to resolve this confusion and shed light on whether the intensification of anticoagulant therapy is recommended. This study identified other *indirect vascular angiopathy signs* on CT pulmonary angiography (CTPA) and involved a novel statistical analysis that was performed to determine the significance of associations between these signs and the CT opacity score of the pathological lung volume, which is calculated by an artificial intelligence system.

## Methods

### Patients

The study was conducted retrospectively, during September and October 2020, with 73 critically ill patients, who were confirmed to have COVID-19 according to RT-PCR and were admitted to the ICU with progressive dyspnea and low O_2_ saturation on room air (PaO_2_ ≤ 93%). They included 53 males and 20 females (73%:27%) and their age ranged from 18 to 88 years (mean ± SD=53.3 ± 13.5). All patients were hospitalized and the patients’ demographic data, medical histories, and laboratory results were collected retrospectively from the hospital information system. Clinical evaluations were performed by a single consulting pulmonologist.

The study was approved by the Ethics Committee of our University Hospital. The need to obtain informed patient consent was waived by the Research Ethics Board with the assurance of the confidentiality of patient information and medical records.

*The inclusion criteria were as follows*: Critically ill patients who were confirmed to have COVID-19 based on RT-PCR and were admitted to the ICU with progressive dyspnea and low O_2_ saturation on room air (PaO_2_ < 93%), which necessitated external airway support with either high-flow nasal oxygen or mechanical ventilation (according to the universal criteria for the clinical classification of COVID-19 patients).

*The exclusion criteria were as follows:* (1) CT images quality was degraded by respiratory motion artifacts, (2) unavailable medical or laboratory records, and (3) cardiomegaly or a history of previous pulmonary vascular disease.

### Imaging modalities

*CT pulmonary angiography (CTPA)* was performed using 64 detector CT scanners from GE Medical Systems (USA) and Siemens SOMATOM Sensation 64 (Germany). The scanning was performed in a supine position with full inspiration and a single breath-hold. The images were obtained in a caudo-cranial direction using the high-quality mode at 120 kVp and 50–100 mAs. The slice thickness was 1.25 mm, and the spacing between slices was 1 mm. The FOV was 350mm × 350 mm. Images were acquired after the IV injection of 60 ml of non-ionic contrast followed by a saline chaser at a flow rate of 5.0–6.0 ml/s using bolus tracking at the threshold of 100 HU on the pulmonary trunk.

### Imaging data analysis

CT images were retrospectively analyzed by three consulting radiologists, who came to a consensus (14–15 years of experience in the field of chest imaging). Images were evaluated in three different greyscale windows: (1) the lung window using a width/level of (1500/−600), (2) the mediastinal window (350/40), and (3) the pulmonary embolism window (700/100). Images were assessed for both morphological and vasculopathy CT signs using minimal intensity projection (MIP) volume reconstruction.

The *morphological signs* included ground-glass opacities (GGOs), consolidations, the crazy-paving pattern (ground-glass opacities with inter-lobular septal thickening), and fibrosis. Extra-pulmonary signs included pleural effusion and mediastinal lymph node enlargement.

The *vasculopathy signs* included the following:

1. Pulmonary embolism which was defined as a contrast filling defect within the pulmonary arterial tree.
2. Pulmonary hypertension which was defined as dilated pulmonary trunk > 3 cm and that exceeded the internal diameter of the nearby portion of the aorta.
3. Pulmonary infarcts which was defined as wedge-shaped sub-pleural ground-glass opacities or consolidative patches.
4. Pulmonary vascular enlargement inside and/or outside the pathological parenchymal ground-glass opacities, which was manifested by asymmetrical internal dilatation of the pulmonary arterial branches. They branches abnormally extended peripherally until the pleural lining, with the loss of normal tapering. Comparison to nearby segments in the same lung or the same segment in the contralateral lung at the same horizontal or axial level was used for assessment [8].
5. The pulmonary vascular “tree-in-bud pattern,” which was identified by dilated, beaded, and branching peripheral pulmonary arterioles that communicated to the pulmonary arterial tree on MIP images [9].

### Quantitative assessment using an artificial intelligence system

Raw CTPA data were automatically transferred from both MDCT machines to a PACS with an incorporated *artificial intelligence system* called the *CT Pneumonia Analysis algorithm.* It was designed by ‘Siemens Health engineers’ to automatically perform quantitative CT measurements in all patients. It did not reside on a specific workstation. This automated quantification of pathological lung volumes is more precise than any other routine human semi-quantitative or qualitative method. It is also essential for standardizing the pathological lung volume calculation between different radiologists.

The artificial intelligence algorithm automatically *identifies* the diseased parts of the lungs (mainly those with high opacity) using an input of 2D CT images and the output of computerized 3D CT images and *quantifies* the pathological lung volumes using 3D segmentation and two major calculations:

1. The opacity score/index was estimated for each lobe of both lungs and then finally summed for both lungs (total opacity score—TOS): score 0 (≤ 25%), score 1 (25–50%), score 2 (50–75%), score 3 (> 75%), and score 4 (100%).
2. The percentage of the predicted volume of abnormal opacity in comparison to the total lung volume was estimated.

Other measurements were also computed such as volume of the lung (ml), opacity volume (ml), high-opacity volume (ml), percentage with opacity within a given lung region (%), total and mean HU of the entire lung and any specific lung region, and the HU standard deviation of the entire lung and any specific lung region. The computerized software calculated all these measurements for both lungs together and per lung, lung lobe and segment.

### Statistical analysis of the data

- The prevalences of abnormal clinical, laboratory, or radiological data (percentage of patients having each criterion or abnormality) were calculated.
- A statistical analysis was performed to determine if there were significant relationship between the radiological CT signs corresponding to pulmonary vascular angiopathy and the automatically calculated CT opacity score. This was performed with chi-squared tests, and the *P* values were obtained with an online calculator (https://www.socscistatistics.com). (A *P* value <0.05 was considered statistically significant.)

## Results

**Fig. 1:**
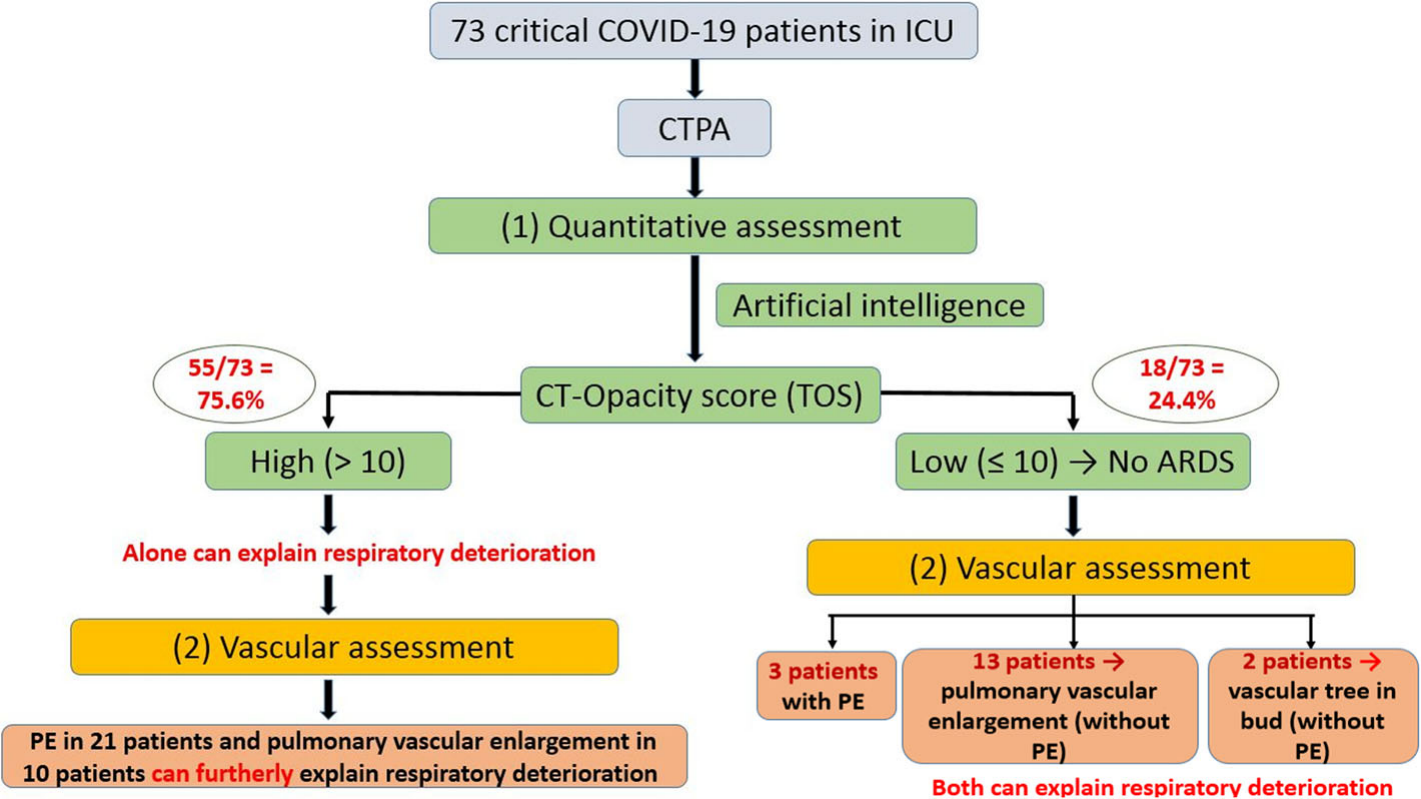
A flowchart is summarizing the steps and results of the study

*A flowchart summarizing the steps and results of the study is shown* *Fig.* *1**.*

### Clinical and laboratory assessments

This retrospective study included 73 critically ill patients with COVID-19: 55 patients (75.3%) had low O_2_ saturation levels on room air (O_2_ sat/RA 80–93%), while 18 patients (24.7%) had even lower O_2_ sat/ RA (<80%). Seventy patients presented after the first week of infection in the first month, while three presented during the second month. D-dimer levels were elevated in 91.8% of the patients. CRP levels were high in 90.4% of the patients. The lymphocyte count was low in 95.9% of the patients. The distributions of patients according to demographic features, clinico-laboratory data, O_2_ requirements, and therapy are fully detailed in Table 1*.*

**Table 1:** Distribution of patients according to demographic features, clinico-laboratory data, O_2_ requirements, and therapy

Demographic features (***N***-%)		Clinical data (***N***-%)		Laboratory profile (***N***-%)		O_**2**_ status and needs (***N***-%)	
**• Age**		**• Duration (time interval)**		**• D-dimer**		**• O**_**2**_ **saturation at room air**	
- <45	*18 (24.7%)*	- 2nd week	*20 (27.4%)*	- Normal	*6 (8.2%)*	- 80%: < 93%	*55 (75.3%)*
- ≥45	*55 (75.3%)*	- 3rd week	*22 (30.1%)*	- High without PE	*43 (58.9%)*	- < 80%	*18 (24.7%)*
- Mean ± SD	*53.3 ± 13.5*	- 4th week	*28 (38.4%)*	- High with PE	*24 (32.9%)*	**• O**_**2**_ **requirements:**	
- (Min.–Max.)	*(18–88)*	- 2nd month	*3 (4.1%)*	- Mean	*1574.1*	- High flow nasal O_2_:	*58 (79.5%)*
				- SD	*1392.2*	- Mechanical ventilation:	*15 (21.5%)*
**• Sex**		**• Symptoms**		**• CRP**			
- Male	*53 (72.6%)*	- Dyspnea	*63 (86.3%)*	- Normal	*7 (9.6%)*		
- Female	*20 (27.4%)*	- Fever	*61 (83.6%)*	- High	*66 (90.4%)*		
		- Cough	*54 (74%)*	- Mean	*7.8*		
		- Chest pain	*8 (11%)*	- SD	*7.3*		
		- GIT-related	*16 (21.9%)*	**• Lymphocytic count**			
		**• Lung co-morbidities**		- Normal	*3 (4.1%)*		
		- Asthma	*4 (66.7%)*	- Low	*70 (95.9%)*		
		- Emphysema	*2 (33.3%)*				
		**• General co-morbidities**					
		- Hypertension	*32 (43.8%)*				
		- Diabetes	*25 (34.2%)*				
		- Lymphoma	*1 (1.4%)*				

### CT opacity score estimated by artificial intelligence

A high CT opacity score (11–20) was observed in 55/73 patients (75.4%), while a low CT opacity score (1–10) was noted in 18/73 patients (24.6%). The detailed distribution of patients according to CT opacity score (either lobar or pulmonary) is also shown in Table 2.

**Table 2:** Distribution of patients according to HRCT findings, CTPA data, and artificial intelligence CT opacity score

HRCT findings		CTPA^**a**^ data		Artificial intelligence CT opacity score:	
❖ **Lobar involvement**		**Pulmonary embolism**		❖ **Right upper lobar**	
- Single	*3 (4.1%)*	❖ **Present or absent**		- Mean	*2.2*
- Multiple	*70 (95.9%)*	- Present	*24 (32.9%)*	- SD^a^	*1.22323*
❖ **Zonal distribution**		- Absent	*49 (67.1%)*	❖ **Right middle lobar**	
- Diffuse	*50 (68.5%)*	❖ **Location**		- Mean	*2.3235294*
- Basal	*19 (26%)*	- Main	*1/24 (4.1%)*	- SD	*1.63062.*
- Upper	*4 (5.5%)*	- Lobar	*7/24 (29.1%)*	❖ **Right lower lobar**	
❖ **Parenchymal changes**		- Segmental	*17/24 (70.5%)*	- Mean	*3.2909091*
- GGO	*67 (91.8%)*	- Sub-segmental	*21/24 (87.5%)*	- SD	*0.9138*
- Crazy-paving	*44 (60.3%)*	❖ **Laterality**		❖ **Left upper lobar**	
- Consolidations	*62 (84.9%)*	- Unilateral	*8/24 (33.3%)*	- Mean	*2.2727273*
- Cysts	*6 (8.2%)*	- Bilateral	*16/24 (66.7%)*	- SD	*1.27609*
❖ **Pleural findings**		**Pulmonary HTN**		❖ **Left lower lobar**	
- Pleural effusion	*6 (8.2%)*	- Positive	*14/73 (19.2%)*	- Mean	*3.0363636*
❖ **Mediastinal findings**		**Pulmonary infarcts**		- SD	*1.29495*
- Enlarged lymph nodes	*19 (26%)*	- Positive	*3/73 (4.1%)*	❖ **Total CT opacity score (20p)**	
- Pneumo-mediastinum:	*1 (1.4%)*	**Vascular dilatation**		⊠ 1–5/20	*4 (5.5%)*
		- Inside (and/or) outside	*57/73 (78.1%)*	⊠ 6–10/20	*14 (19.2%)*
		^*a*^ High TOS^a^ (46-81%), ^a^ low TOS (11–19%)		⊠ 11–15/20	*28 (38.4%)*
		- Inside (+) outside	*40/73 (54.8%)*	⊠ 16–20/20	*27 (37%)*
		^*a*^ High TOS (33–72%), ^a^ low TOS (7–18%)			
		- Pleural extension	*45/73 (61.6%)*		
		^*a*^ High TOS (40–89%), ^a^ low TOS (5–11%)			
		**Vascular “tree in bud”**			
		- Positive	*10/73 (13.7%)*		

^a^ *CTPA* CT pulmonary angiography, *TOS* total opacity score, *SD* standard deviation

### CTPA findings

**Fig. 2:**
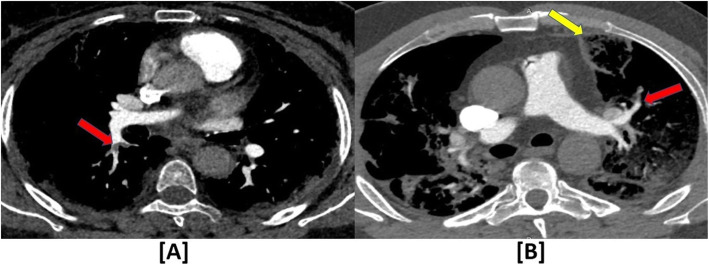
COVID-19 induced acute pulmonary embolism and pulmonary infarction. **a** A 66-year-old female patient with *acute pulmonary embolism (PE)*; axial mediastinal window CTPA revealed a small branching filling defect at the right lower lobar pulmonary artery (red arrow). **b** A 52-year-old male patient with acute pulmonary embolism (PE) and *pulmonary infarction (PI)*; axial mediastinal window CTPA revealed a small filling defect at the anterior segmental branch of the left upper lobar pulmonary artery (red arrow) and sub-pleural wedge-shaped lesion showing bubbly air densities (yellow arrow)

**Fig. 3:**
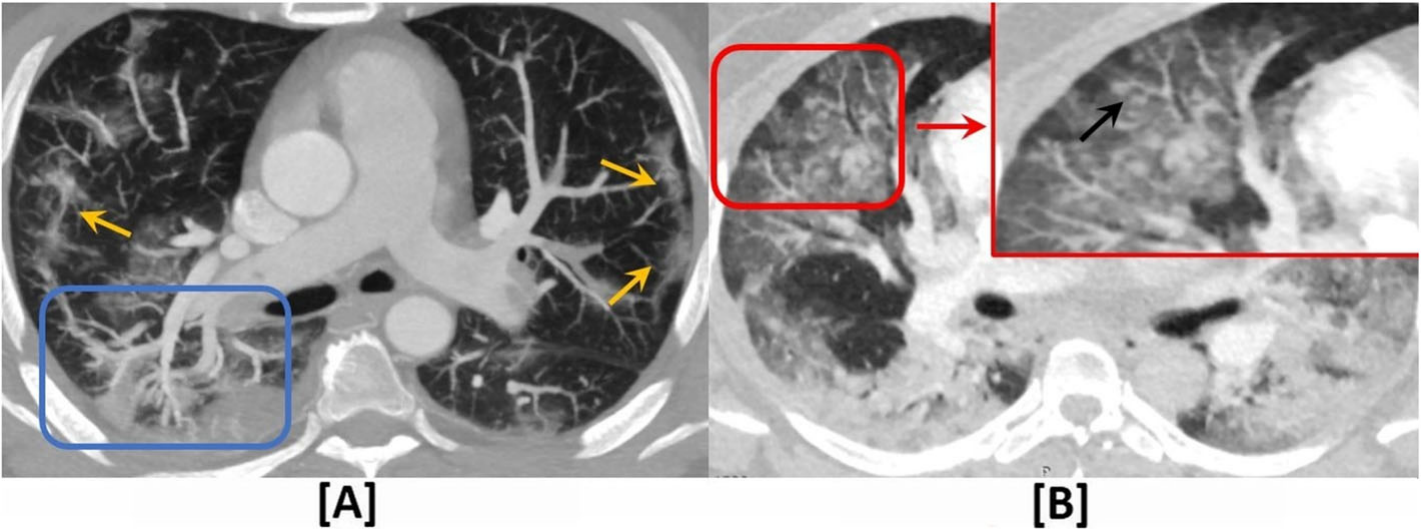
Indirect CTPA signs of COVID-19-induced pulmonary vascular angiopathy. **a** A 43-year-old male patient with *pulmonary vascular enlargement (PVE)*; axial lung window CTPA showed asymmetrical dilated right lower lobar pulmonary arterial branches (blue square) together with peripheral sub-pleural ground glass patches (orange arrows). **b** A 37-year-old female patient with *vascular tree-in-bud pattern (TIB)*; axial zoomed lung window CTPA showed beaded and branching distal pulmonary arterioles

Five radiological signs consistent with pulmonary vasculopathy were observed (Figs. 2 and 3): (1) pulmonary vascular enlargement (inside and/or outside the parenchymal opacities), which was observed in 57/73 patients (78.1%); (2) pulmonary embolism, which was observed in 24/73 patients (32.9%); (3) Pulmonary hypertension, which was observed in 14/73 patients (19.2%)*;* (4) vascular “tree-in-bud pattern,” which was observed in 10/73 patients (13.7%); and (5) pulmonary infarction, which was observed in only 3/73 patients (4.1%). The detailed distribution of patients according to these CTPA findings is also shown in Table 2.

### HRCT findings

The most common parenchymal CT changes were ground-glass opacities (GGOs), which were observed in 91.8% of the patients. Consolidative changes and the crazy-paving pattern were observed in 84.9% and 60.3% of the patients, respectively. The detailed distribution of patients according to these HRCT findings is presented in Table 2.

### Statistical results (Table 3)

#### Pulmonary vascular angiopathy CTPA signs

**Fig. 4:**
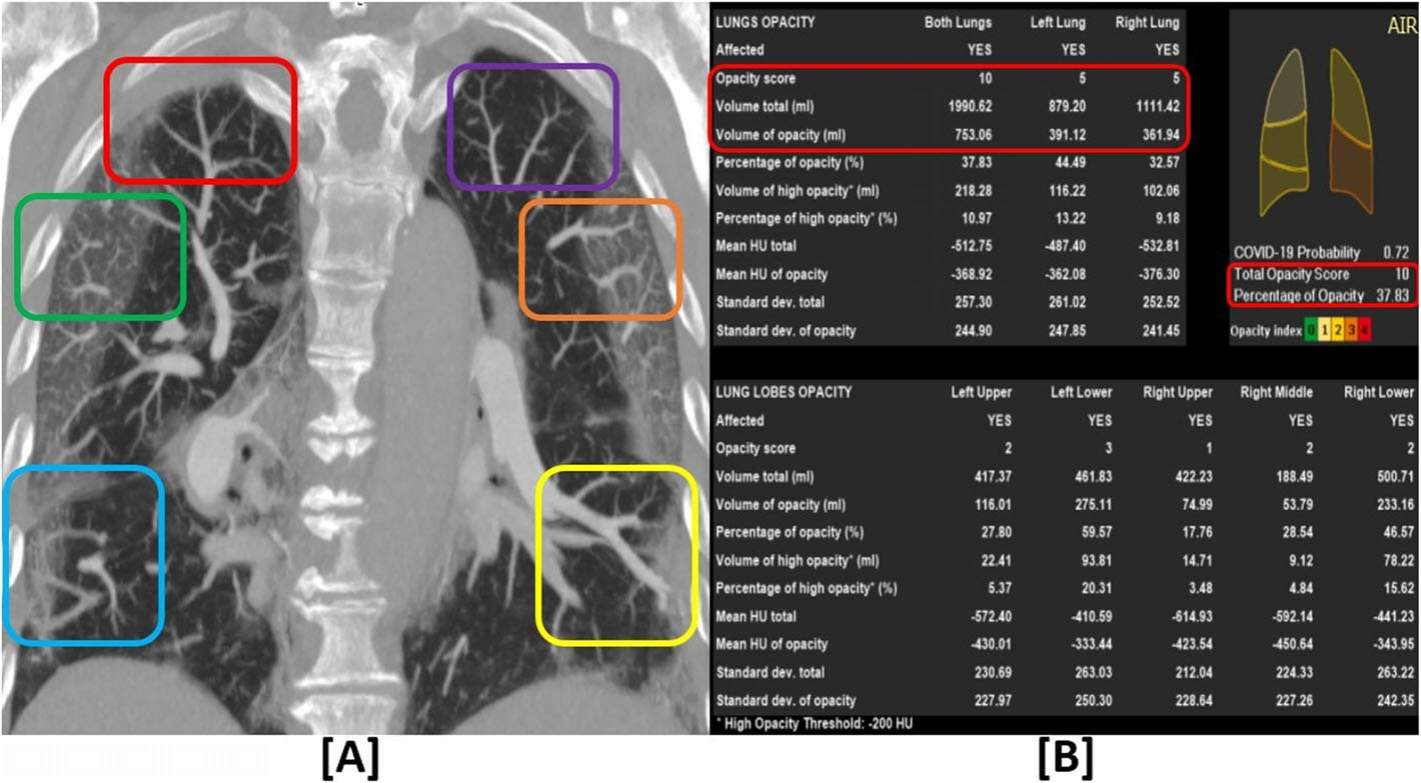
A 72-year-old COVID-19 male patient with pulmonary vascular enlargement (PVE) on a background of borderline total opacity score (TOS): **a** Coronal lung window CTPA revealed bilateral basal pulmonary vascular dilatation (more on the left side with relatively increased opacity density) manifested by abnormally increased calibre within the pulmonary abnormal opacity as well as lack of tapering and extension till the pleural surface (blue and yellow squares). Bilateral upper lobar PVE is also noted within the pulmonary abnormal ground-glass opacity (more on the left side—brown square) compared to the nearby cranial vessels in (purple square) and contralateral same level vessels in (green square). Right apical PVE outside the abnormal opacity is also noted (red square). **b** Detailed *artificial intelligence* quantitative assessment revealed: *TOS = 10/20 and volumetric percentage = 37.83%*

**Fig. 5:**
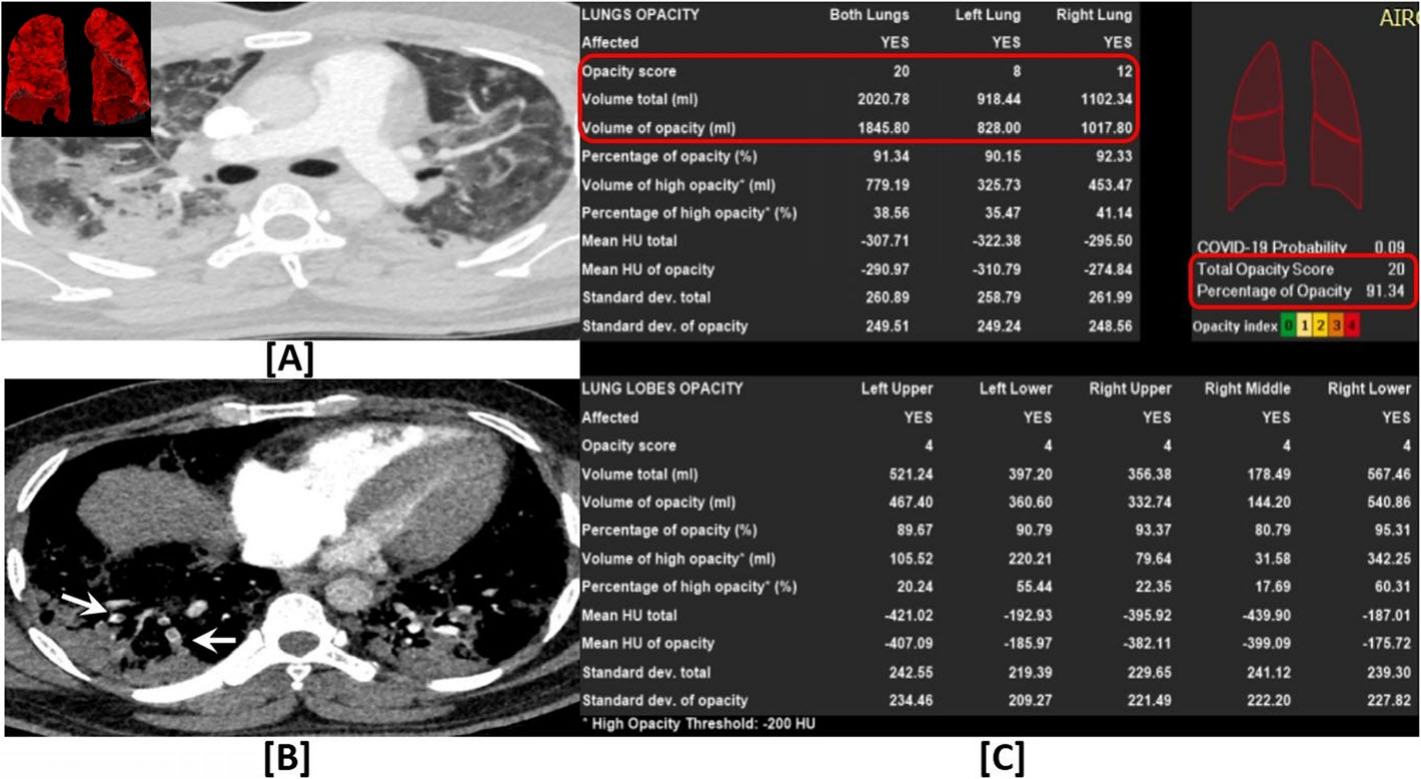
A 34-year-old COVID-19 male patient with acute pulmonary embolism (PE) on a background of high total opacity score (TOS); **a** Axial lung window CTPA showing bilateral widespread large ground-glass opacities and consolidative changes. **b** Axial mediastinal window CTPA showing right lower lobar multiple segmental and sub-segmental acute pulmonary emboli (PE) (white arrows). **c** Detailed *artificial intelligence* quantitative assessment revealed: *TOS = 20/20 and volumetric percentage = 91.34%*

Pulmonary vascular enlargement was found in 13/15 patients (86.7%) who had low CT opacity scores and did not have pulmonary embolism. The vascular “tree-in-bud” sign was also found in 2/15 of these patients (13.3%). Pulmonary hypertension and pulmonary infarctions were not identified in any of these patients (Figs. 4 and 5*)*. There were no significant associations of these CTPA signs with the CT opacity scores (*P* values 0.3205–0.7551 all >0.05).

**Table 3:** Distribution of patients with vascular angiopathy and morphological CT signs according to low versus high TOS and negative versus positive pulmonary embolism with significant statistical analysis

Radiological signs			Low TOS (≤ 10) (***N***^**a**^ = 18 patients)		High TOS (> 10) (***N*** = 55 patients)	
			Negative PE^**a**^	Positive PE (***N***=3)	Negative PE	Positive PE (***N***=21)
**Vascular angiopathy CTPA signs**						
**1.**	**Pulmonary “vascular enlargement”**	Present (*T*=57)	13	0	34	10 (overlap)
		Absent	2	3	0	11
		*X*^2a^	0.4794			
		*P* value	0.488706 (>0.05) → No significant relation to TOS			
**2.**	**Pulmonary hypertension**	Present (*T*=14)	0	3 (overlap)	0	11 (overlap)
		Absent	15	0	34	10
		*X*^2^	0.0972			
		*P* value	0.755197 (>0.05) → No significant relation to TOS			
**3.**	**Vascular “tree in bud”**	Present (*T*=10)	2	0	8	0
		Absent	13	3	26	21
		*X*^2^	0.1353			
		*P* value	0.712997 (>0.05) → No significant relation to TOS			
**4.**	**Pulmonary infarction**	Present (*T*=3)	0	0	0	3 (overlap)
		Absent	15	3	34	18
		*X*^2^	0.9868			
		*P* value	0.3205 (>0.05) → No significant relation to TOS			
**Other morphological CT signs:**						
**1.**	**Crazy-paving**	Present (*T*=44)	6	2	18	18
		Absent	9	1	16	3
		*X*^2a^	2.5001			
		*P* value	0.1138 (>0.05) → No significant relation to TOS			
**2.**	**Consolidations**	Present (*T*=62)	12	3	34	13
		Absent	3	0	0	8
		*X*^2a^	0.0477			
		*P* value	0.8271 (>0.05) → No significant relation to TOS			
**3**	**Pleural effusion**	Present (*T*=6)	0	1	1	4
		Absent	15	2	33	17
		*X*^2a^	0.2247			
		*P* value	0.6355 (>0.05) → No significant relation to TOS			
**4.**	**Enlarged L.Ns**	Present (*T*=19)	0	1	15	3
		Absent	15	2	19	18
		*X*^2a^	4.6925			
		*P* value	0.0303 (<0.05) → Significant relation to TOS			

^*a*^*TOS* total opacity score*, N* total number of patients*, PE* pulmonary embolism*, X*^*2*^ chi-squared statistical test*, P value* statistical significant relation when < 0.05

#### Morphological CT signs

The crazy-paving pattern was identified in 6/15 patients (40%) who had low CT opacity scores and no pulmonary embolism. Consolidation pattern was also found in 12/15 of these patients (80%). Pleural effusion and significant nodal enlargement were not identified in any of these patients. A significant association was identified between lymph node enlargement and the CT opacity score, with a *P* value of 0.0303 (<0.05) (Table 3).

### Impact on clinical management

All included patients had already been admitted to the ICU. A total of 58/73 patients (79.5%) received high-flow nasal oxygen therapy. In total, 15/73 patients (21.5%) needed mechanical ventilation: all of them (100%) had the crazy-paving pattern, 10 patients (67.7%) had pulmonary vascular enlargement, and one patient (6.7%) had a pulmonary vascular tree-in-bud pattern.

All patients initially received dexamethasone, immunomodulator drug therapy, and thromboprophylaxis agents. Based on the CTPA results, therapeutic doses of anticoagulant therapy were prescribed not only for those 24 patients with acute pulmonary embolism but also for the other 47 patients who had pulmonary vascular enlargement without acute pulmonary embolism.

## Discussion

The primary cause of mortality related to COVID-19 is respiratory failure. However, the pathophysiology of respiratory failure in patients with COVID-19 differs from the “conventional” ARDS caused by other infectious diseases. Emerging evidence from recent autopsies has shown widespread vasculopathy associated with COVID-19. Furthermore, several patients showed progressive deterioration of their respiratory function that could not be explained by the extent of parenchymal lung changes. This fact directed attention to the emerging evidence of widespread vascular pathology and thromboembolic events associated with COVID-19 [9].

Approximately 75% of the critically ill patients in this study had high CT opacity scores with widespread ground-glass opacities, fibro-consolidative changes, and the crazy-paving pattern (radiological signs that indicate ARDS). This matched the findings in the study by Raptis CA et al. [10]. However, the respiratory deterioration in the remaining 25% of the patients who had low CT opacity scores did not have a clear clinical cause.

This study found varying pulmonary vascular angiopathic signs on CTPA in these patients with COVID-19, approximately 1–4 weeks after the primary infection.

In particular, “pulmonary vascular enlargement” was the most common radiological finding. This is consistent with findings in the study by Bai et al. [11], in which pulmonary vascular thickening or enlargement was identified in 59% of their COVID-19 patients but in only 22% of patients with other types of viral pneumonia; the difference was significant (<0.001). Li et al. [12] and Caruso et al. [13]—also found this sign in 82.4% and 89% of their patients, respectively. These results were also similar to those in the study by Lang et al. [8] in which dilated pulmonary vessels were identified in 85% of the patients: 79% were identified within the pulmonary opacities, and 56% were identified outside of lung opacities. Additionally, the dilated distal pulmonary vessels extended to the pleura in 83% of the cases. They attributed pulmonary vascular dilatation to the failure of normal hypoxic pulmonary vasoconstriction and dysfunctional vasoregulation. This typically is a result of the diffuse inflammatory process and the consequent over-activation of the regional vasodilatation cascade. This can explain COVID-19-related silent hypoxia in patients with normal lung compliance who develop a significant ventilation/perfusion mismatch.

The results of this study matched those of the study by Leonard-Lorant et al. [14], in which acute PE was identified in 30% of the patients. This proportion was even higher than the proportion of the critically ill patients without COVID-19 (1.3%) and the proportion of emergency department patients (3–10%).

“The vascular tree-in-bud pattern” is a newly described sign that was defined in our study as dilated branching sub-segmental pulmonary arterioles that were not obscured by dense parenchymal opacification. This was consistent with the definition used in the study by Fox et al. [15], in which these enhanced distal pulmonary branches were assumed to be a sign of pulmonary thrombotic micro-angiopathy as supported by previous postmortem data. This pattern was identified in 13.7% of our patients, which was a much smaller proportion than the 64% reported in the study by Patel et al. [16]. Pulmonary infarctions and pulmonary hypertension were also described in our study, which was similar to the findings in the study by Lang et al. [8]

There were no significant relationships identified between these vasculopathy CT signs and the CT opacity scores; therefore, the presence of pulmonary vascular enlargement and, less commonly, the vascular tree-in-bud sign can explain the deterioration in respiratory condition among those patients with a low CT opacity score and no pulmonary embolism.

The strength of this study in comparison with the previous literature was the analysis of correlations between the described pulmonary vascular angiopathic CT findings and the CT opacity score obtained with an artificial intelligence algorithm, which was performed to elucidate the reason for the progressive deterioration in respiratory condition among patients without CT signs of “white lung” or acute pulmonary embolism.

This study was limited by the small sample size, which was due to the low percentage of patients with critical COVID-19.

## Conclusion

Every radiologist should be familiar with five signs of COVID-19 induced pulmonary vascular angiopathy on CT namely, pulmonary vascular enlargement, pulmonary embolism, pulmonary hypertension, pulmonary vascular “tree-in-bud pattern,” and pulmonary infarction. The presence of “pulmonary vascular enlargement” and less commonly the “vascular tree-in-bud” signs on CTPA can explain the respiratory deterioration in patients who do not have COVID-19 without severe parenchymal involvement, also called “white lung” or acute pulmonary embolism. The presence of the crazy-paving pattern can also explain this confusing clinical result.

## Data Availability

The datasets used and/or analyzed during the current study are available from the corresponding author on reasonable request.

## Abbreviations

COVID-19: Coronavirus disease 2019
RT-PCR: Reverse transcription-polymerase chain reaction
CTPA: Computed tomography pulmonary angiography
PE: Pulmonary embolism
TOS: Total opacity score
